# Workload and procedures used by European data protection authorities related to personal data protection: a cross-sectional study

**DOI:** 10.1186/s13104-023-06308-z

**Published:** 2023-03-27

**Authors:** Livia Puljak, Anamarija Mladinić, Zvonimir Koporc

**Affiliations:** 1 Center for Evidence-Based Medicine and Health Care, Catholic University of Croatia, Ilica 242, Zagreb, 10000 Croatia; 2 Croatian personal data protection agency (AZOP), Selska cesta 136, Zagreb, Croatia

**Keywords:** Personal data protection, European Union, Data protection authorities

## Abstract

**Objective:**

Data protection authorities (DPAs) are independent public authorities supervising the application of the data protection law. There is one DPA in each European Union (EU) Member State. Workload and procedures used by European DPAs were analyzed via a cross-sectional study.

**Results:**

DPAs from 13 countries participated: Austria, Bulgaria, Croatia, Estonia, Finland, Greece, Italy, Latvia, Liechtenstein, Lithuania, Norway, Romania, and Slovakia. Responding to opinion/guidance requests in DPAs was highly heterogeneous. Procedure types used by DPAs varied, from telephone-based advisory service in Norway to a formal legal opinion in Austria. The deadline for responding to the requests varied considerably in DPAs. The number of opinion/guidance requests sent by data controllers and processors, and the number of opinion/guidance requests and complaints sent by data subjects, increased from 2015 to 2018 when the General Data Protection Regulation (GDPR) came into full effect; it decreased in 2019. Few DPAs organized education about data protection for the research community. In conclusion, the procedures and workload of DPAs in the EU were highly variable. It is important to study these aspects further, as they may assist in tailoring future data protection policies and procedures at the EU level.

**Supplementary Information:**

The online version contains supplementary material available at 10.1186/s13104-023-06308-z.

## Introduction

Data protection authorities (DPAs) are independent public authorities whose task is to supervise the application of the data protection law [1]. DPAs provide expert advice on data protection issues and handle complaints filed against violations of the General Data Protection Regulation (GDPR) [2] and the relevant national laws. Very few research studies can be found on the topic of data protection in the EU. Recently, we published a survey among data protection officers (DPOs), examining the scope of work, type of work, and education of DPOs in institutions in Croatia [3]. However, when searching for studies on DPAs, we were unable to find any research reports that analyzed how different DPAs handle issues and complaints regarding data protection and their workload before and after the introduction of GDPR.

GDPR came into effect in May 2018 [2]. The implementation of the GDPR led to the improvement of personal data protection. Also, it strongly affected the research within the EU [4–6]. In that context, the pivotal role of DPOs in data protection has been clearly recognized and their autonomy and independence need to be further strengthened [7]. We have previously shown that DPOs have expressed that their work burden has increased after the GDPR enforcement [3].

We hypothesized that the number of data protection issues and handled complaints filed against violations of the GDPR will increase in the DPAs post-GDPR enforcement. However, some specific differences within national legislations among EU member states, as well as the support which each DPA receives from their governmental institutions, variations in procedures, prescribed deadlines for response to requests together with differences in the number of employees and experts involved in their work might have a strong effect on the GDPR implementation procedures.

In September 2022, European Data Protection Board (EDPB) published a report on the resources made available by the Member States to the data protection supervisory authorities. In the report, 77% of DPAs from the European Economic Area (EEA) explicitly stated that they do not have enough financial resources. Furthermore, 87% claimed they do not have enough human resources to carry out their activities. From the report, it can be concluded that this is mainly due to a significant increase in the number of complaints filed by individuals to DPAs [8].

Due to the lack of data, this study aimed to analyze the workload and procedures used by European DPAs related to personal data protection.

## Methods

### Study design

A cross-sectional study was conducted.

### Setting

For this study, a new survey was designed because literature search did not yield any survey on this topic. The authors involved in the survey design were experts in data protection and research methodology.

The study was conducted among DPAs from European countries belonging to the EEA, which includes 27 EU member states, as well as Iceland, Liechtenstein and Norway. These countries were chosen because GDPR applies to the member states of EU and all countries in the EEA.

The survey used in the study was sent to DPAs via e-mail by the author AM, from her official e-mail address of the Croatian Agency for Personal Data Protection (AZOP). The e-mail invitation to participate in the study, together with information about the study, was sent on June 2, 2020; data collection was closed in July 2021. The DPAs received up to 4 reminders spaced 3 months apart, if they did not respond.

### Participants

The participants were contact persons representing DPAs; one person for each DPA. Representatives of all European DPAs to participate in the study were invited.

### Questionnaire

For the purpose of this study, a new questionnaire for DPAs was designed due to lack of availability of such questionnaires in the literature. The questionnaire is available in Supplementary file 1. Three authors designed the first version of the questionnaire – a data protection officer, research ethics expert, and methodologist. Draft of the questionnaire was further circulated among additional data protection experts from the Croatian DPA, for instrument pretesting and to create a final version of the questionnaire.

The questionnaire contained 14 questions (Supplementary file 1) regarding the procedures used by DPAs for responding to opinion/guidance requests, handling complaints of citizens, the workload in terms of different procedures handled before and after the GDPR implementation, number of opinion/guidance requests, and complaints for scientific research and a non-medical type of research; the number of cases/complaints that went to the court, provision of training/education for different target audiences, including research community. All questions were open-ended. Scoring methods were not used.

We did not calculate Cronbach’s alpha for this questionnaire because each item represented a unique context rather than an underlying latent construct.

### Data analysis

For responses that were not numerical, each response was categorized using a codebook that was not defined *a priori*. Descriptive statistics was used to calculate the frequency and percentage of responses.

### Reporting

The study was reported in line with the STROBE checklist [9]. The STROBE checklist for this manuscript is available in Supplementary file 2.

## Results

Responses to the survey questions were received from DPAs in the following 13 countries: Austria, Bulgaria, Croatia, Estonia, Finland, Greece, Italy, Latvia, Liechtenstein, Lithuania, Norway, Romania, Slovakia. One agency, from Slovenia, responded that they have no resources to provide the data asked in the survey. Response rate was 43% (13/30 invited countries).

The procedure of responding to opinion/guidance requests in DPAs was highly heterogenous and not standardized on the EU level (Table 1). Procedure types used by DPAs were variable, from simple telephone-based advisory service in Norway, to a formal legal opinion in Austria. The deadline to respond ranged from within 30 days to the maximum of 14 weeks. Some agencies had an option to extend this deadline to unspecified time point (Table 1).

**Table 1:** The procedure of responding to opinion/guidance requests in data protection agencies

Data protection agency	Type of procedure	Regulation	Deadline
Austria	Legal opinions can only be provided in a formal complaint procedure	Articles 52 and 77 of GDPR	Not reported
Bulgaria	Answer	Not reported	Within one month; the deadline can be extended depending on the matter
Croatia	Expert opinion	Act on Implementation of GDRP	30 days from the day of submission; if necessary to involve other bodies in the country or abroad, may be extended for another 30 days
Estonia	Not specified	Estonian Response to Memoranda and Requests for Explanations and Submission of Collective Proposals Act	30 days (can be extended to 60 days)
Finland	Answer	The Administrative Procedure Act (*434/2003)* Act on the Openness of Government Activities (*621/1999*)	Not specified (“without undue delay”)
Greece	No obligation to answer the question that do not fall under the provisions of the GDPR	Article 57 of GDPR	There are no legal deadlines for providing answer.
Italy	Opinion following prior consultation following a data protection impact assessment	Article 36, paragraph 1, of the GDPR and Sect. 2-p and 110, paragraph 1 of Legislative Decree no. 196/2003	8 weeks after receipt of the request, which can be extended by a further 6 weeks.
	Opinions on proposals for legislative measures or regulatory measures based on such legislation	Article 36, paragraph 4, of the GDPR and Sect. 154, paragraph 5, of Legislative Decree no. 196/2003	45 days after receipt of the request, without prejudice to shorter periods provided for by law.
	Opinion on medical, biomedical and epidemiological research programmes and projects	Article 36, paragraph 1, of the GDPR and Sect. 110, paragraph 1 of Legislative Decree no. 196/2003.	8 weeks after receipt of the request, which may be extended by a further 6 weeks.
Latvia	Not specified	Administrative Procedure Law. Regarding opinion/guidance requests, Inspectorate bases on Article 98 of the Law	Within 30 days
Liechtenstein	Answers	Not reported	There is no precise deadline
Lithuania	Request for consultation	Article 10 of Law of Republic of Lithuanian on public administration	20 working days
Norway	Telephone-based advisory service	Not reported	Not reported
Romania	No specific procedure	Ordinance no. 27/2002 on the regulation of the activity of solving petitions.	Not reported
Slovakia	Answer/consultation	GDPR	There are no legal deadlines for providing answer.

Procedure for handling the complaints of the citizens were described as a free-form application or an electronic form; while some DPAs only cited applicable national law or GDPR. Deadlines for handling the citizens’ complaints range from 30 days to 9 months. In Liechtenstein, there is no precise deadline at all (Supplementary file 3; Supplementary Table 2).

The majority of DPAs have multiple options of dealing with the complaints, where the mediations was the most common option. Most of the DPAs did not have a prespecified maximum response time for such other options (Supplementary file 3 ; Supplementary Table 3).

**Fig. 1:**
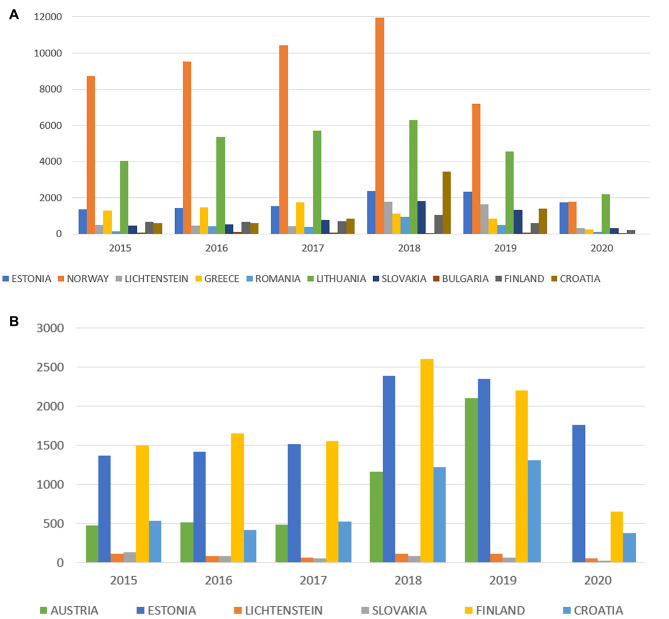
(**A**) The number of opinion/guidance requests sent by data controllers and processors regarding compliance with the data protection legal framework for years provided by 10 countries. (**B**) The number of opinion/guidance requests and complaints sent by data subjects for years, provided by 6 countries

The number of opinion/guidance requests sent by data controllers and processors regarding compliance with the data protection legal framework for years provided by 10 countries is shown in Fig. 1A. The number of opinion/guidance requests and complaints sent by data subjects for years, provided by 6 countries, is shown in Fig. 1B. As shown in Fig. 1A and B, the number of those requests or complaints increased from 2015 to 2018 when the GDPR came into full effect, and then it decreased in 2019.

Seven DPAs provided numbers of opinion/guidance requests and complaints regarding personal data protection related specifically to scientific research they received in the period from January 1, 2015 to May 1, 2020. These numbers were very low, ranging from 0 in Latvia to 132 in Finland (Supplementary file 3; Supplementary Table 6). Among those, requests and complaints regarding non-medical research in the same period ranged from 0 in Latvia to 34 in Croatia. Multiple DPAs did not record such data (Supplementary file 3; Supplementary Table 7).

The number of cases/complaints reported by the citizens related to violation of their right to personal data protection that went to the court (official misdemeanor proceedings) in the period from January 1, 2015 to May 1, 2020 ranged from 23 in Slovakia to 411 in Italy (Supplementary file 3; Supplementary Table 8).

Related to the previous question, regarding complaints related to scientific research from January 1, 2015, to May 1, 2020, that went to the court (official misdemeanor proceedings) ranged from 0 in Norway to 20 in Italy. However, the majority of DPAs did not keep records about such events 8 (Supplementary file 3; Supplementary Table 9).

Nine DPAs responded that they organize GDPR training sessions/education. The audience for those education were DPOs, data controllers, data processors, small and medium enterprises (SMEs), various authorities, lawyers, legal professions, general public, public or private sector, children. When describing these audiences, only Greece reported that they organize education for researchers (Table 2) (Supplementary file 3; Supplementary Tables 10 and 11).

**Table 2:** Organization of GDPR training sessions/education and the audience

Data protection agency	Organizing GDPR training sessions/education?	For whom
Austria	No	Not applicable
Bulgaria	Yes	DPOs
Croatia	Yes	-SMEs -DPOs from all sectors -Children
Estonia	Yes	-Data subjects -Data controllers -Data processors -Media
Finland	Yes	-General public -Various authorities -Companies -DPOs
Greece	Yes	-DPOs -Civil servants -Lawyers -General public -Researchers -Children
Italy	Yes	-DPOs -SMEs -Legal professions
Latvia	Yes	-Public sector -Private sector -Children -SMEs
Liechtenstein	Yes	-Communal authorities -DPOs -Associations -General public -Students and their parents -Certain groups of professionals
Lithuania	Yes	-DPOs -Journalists -Start-ups -Representatives of healthcare services -SMEs -Vulnerable society groups -Youth -Seniors
Norway	No	Not applicable
Romania	No	Not applicable
Slovakia	No	Not applicable

Acronyms: DPO = data protection officer, SME = small and medium enterprises

When asked specifically do they organize GDPR training sessions /education for the scientific research community, only Bulgaria responded that they organize education for such audience (Supplementary file 3; Supplementary Table 12). When asked how often do they provide such training/education and how many individuals usually attend such training/education, Bulgarian DPA responded “about 1 per year with about 25–50 participants” (Supplementary file 3; Supplementary Table 13).

## Discussion

GDPR came into effect in May 2018 [10], bringing significant changes in the area of personal data protection across the EU that strongly affected different areas of our life [11–14].

Some recent studies showed differences in enforcement of GDPR among member states [15], as well as the need for better standardization of DPAs’ procedures in the area of fines prescription [16]. Aiming to analyse differences in the workload and procedures used by European DPAs related to personal data protection, this study found that the procedures and workload of DPAs in the EU were highly variable.

It needs to be emphasized that each country has its own laws, and legally prescribed procedures and deadlines. Thus, it may be challenging to expect that each European country will align its national laws in this respect. For national laws, it is important that they are aligned with the GDPR in a way that the national laws do not include regulations that are contrary to the GDPR.

Also, there is no uniformity in terms of reporting statistics regularly collected by different DPAs. For example, the survey asked for a number of opinions/guidance requests, and also for a number of complaints received in the analyzed period. Some countries provided feedback that they do not, for example, keep records about the number of opinions/guidance requests.

Some discrepancies were observed in the data received by DPAs. On the question about the audiences for which the DPAs organize their training, DPA from Greece was the only one that mentioned researchers as the targeted audience of their training. When asked specifically whether they organized GDPR training for the scientific research community, only Bulgaria responded that they organize education for such audience. Overall, it appears that few DPAs recognize researchers as the targeted audience in need of GDPR training.

Furthermore, few DPAs provided information on data protection issues that involved research topics. This is in line with our previous study, in which we have shown that very few research-related requests were received by the Croatian DPA both before and after the enforcement of the GDPR [17]. As GDPR stipulates, the burden of aligning with the GDPR lies with the data controllers [2]. It is important to foster interest among research institutions and universities to invest in education about data protection, and to educate researchers. This is particularly relevant in the context of the research and innovation area for Europe’s future [18].

## Conclusion

In conclusion, the procedures and workload of DPAs in the EU were highly variable. It is important to study these aspects further, as they may assist in tailoring future data protection policies and procedures at the EU level.

### Limitations

The limitations of this study include cross-sectional nature of the study. Longitudinal study of DPAs would better describe any changes that were adopted by the DPAs in their procedures, to depict their evolution. Furthermore, 13 DPAs responded to our survey invitation, which provides partial information about DPAs in the EU (non-responder bias). Thus, our results cannot be generalized to the entire EU.

## Electronic supplementary material

Below is the link to the electronic supplementary material.

Supplementary Material 1

Supplementary Material 2

Supplementary Material 3

## Data Availability

All raw data collected within the study are reported in Supplementary file 3.

## List of abbreviations

AZOP: Croatian Agency for personal data protection
DPA: Data Protection Agency
DPO: Data Protection Officer
EEA: European Economic Area
EU: European Union
GDPR: General Data Protection Regulation
