# Supplementary material for: Workload and procedures used by European data protection authorities related to personal data protection: a cross-sectional study
Source: BMC Res Notes. 2023 Mar 27;16:41. doi: 10.1186/s13104-023-06308-z (PMC10045515; doi:10.1186/s13104-023-06308-z)
Supplement: Supplementary file 1 — Supplementary Material 1 [file 13104_2023_6308_MOESM1_ESM.docx]

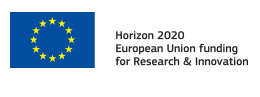

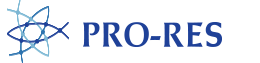


Dear Colleagues,

Together with researchers from the European H2020 project PRO-RES

(<http://prores-project.eu/>) Croatian DPA is conducting a survey among data protection authorities in EU.

This survey is part of the larger study, in which we are analyzing procedures related to personal data protection in medical and non-medical research.

We would appreciate if you could provide us the following information regarding processes in your authority:

1. What is the procedure of responding to **opinion/guidance requests** in your authority regarding compliance with GDPR and data protection legal framework, and in which deadline?

2. What is the procedure of handling the **complaints** of the citizens, and in which deadline?

3. Are there multiple options of dealing with the **complaints** (amicable resolution, mediation) and is there a prespecified maximum response time?

4. If it is possible, please provide us the number of **opinion/guidance requests** sent by **data controllers and processors** regarding compliance with the data protection legal framework for years:

2015

2016

2017

2018 (in the period from January 1, 2015, to May 24, 2018, pre-GDPR period)

2018 (in the period May 25, 2108, to December 31, 2018, post-GDPR period)

2019

2020 (until May 1, 2020?)

5. If it is possible, please provide us the number of **opinion/guidance requests and complaints** sent by **data subjects** for years:

2015

2016

2018 (in the period from January 1, 2015, to May 24, 2018)

2018 (in the period May 25, 2108, to December 31, 2018)

2019

2020 until May 1, 2020?

6. How many **opinion/guidance requests and complaints** regarding personal data protection related **specifically to scientific research** your authority received in the period from
January 1, 2015 to May 1, 2020?

7. Regarding **opinion/guidance requests** and **complaints** related to scientific research, how many of those reported from January 1, 2015 to May 1, 2020 were related specifically to **non-medical** research?

8. Could you please provide us information on how many **cases/complaints reported by the citizens related to violation of their right to personal data protection** went to the court (official misdemeanor proceedings) in the period from January 1, 2015 to May 1, 2020?

9. Related to the previous question, regarding **complaints** related to **scientific research**, how many of those reported from January 1, 2015, to May 1, 2020, went to the court (official misdemeanor proceedings)?

10. Does your authority organize GDPR training sessions/education?

Yes/No

11. If yes, who is the target audience of such GDPR training sessions /education?

12. Does your authority organize GDPR training sessions /education for the scientific research community?

13. If your authority does organize GDPR training/education for the scientific research community, how often do you provide such training/education and how many individuals usually attend such training/education?

Thank you very much for sharing this information with us. We intend to present data received from DPAs in a research report about the overall study that the PRO-RES project is conducting.
