# Supplementary material for: Workload and procedures used by European data protection authorities related to personal data protection: a cross-sectional study
Source: BMC Res Notes. 2023 Mar 27;16:41. doi: 10.1186/s13104-023-06308-z (PMC10045515; doi:10.1186/s13104-023-06308-z)
Supplement: Supplementary file 2 — Supplementary Material 2 [file 13104_2023_6308_MOESM2_ESM.doc]

STROBE Statement—Checklist of items that should be included in reports of ***cross-sectional studies***

|  | Item No | Recommendation | Check for this manuscript |
| --- | --- | --- | --- |
| **Title and abstract** | 1 | (*a*) Indicate the study’s design with a commonly used term in the title or the abstract | *The type of study is indicated in the*  *title* |
|  |  | (*b*) Provide in the abstract an informative and balanced summary of what was done and what was found | *The abstract gives a summary of the*  *study* |
| Introduction | | |  |
| Background/rationale | 2 | Explain the scientific background and rationale for the investigation being reported | *Background and rationale are*  *reported* |
| Objectives | 3 | State specific objectives, including any prespecified hypotheses | *Aims are detailed in the Introduction* |
| Methods | | |  |
| Study design | 4 | Present key elements of study design early in the paper | *Key elements are reported* |
| Setting | 5 | Describe the setting, locations, and relevant dates, including periods of recruitment, exposure, follow-up, and data collection | *Setting is described* |
| Participants | 6 | (*a*) Give the eligibility criteria, and the sources and methods of selection of participants | *Eligibility criteria are described* |
| Variables | 7 | Clearly define all outcomes, exposures, predictors, potential confounders, and effect modifiers. Give diagnostic criteria, if applicable | *Outcomes are described.*  *Other items: not applicable* |
| Data sources/ measurement | 8* | For each variable of interest, give sources of data and details of methods of assessment (measurement). Describe comparability of assessment methods if there is more than one group | *Data sources are described* |
| Bias | 9 | Describe any efforts to address potential sources of bias | *Non-responder bias is mentioned in Limitations* |
| Study size | 10 | Explain how the study size was arrived at | *This is reported – all eligible agencies were invited* |
| Quantitative variables | 11 | Explain how quantitative variables were handled in the analyses. If applicable, describe which groupings were chosen and why | *Statistical analysis is reported* |
| Statistical methods | 12 | (*a*) Describe all statistical methods, including those used to control for confounding | *Statistical methods are reported* |
|  |  | (*b*) Describe any methods used to examine subgroups and interactions | *Not applicable* |
|  |  | (*c*) Explain how missing data were addressed | *Not applicable* |
|  |  | (*d*) If applicable, describe analytical methods taking account of sampling strategy | *Not applicable* |
|  |  | (*e*) Describe any sensitivity analyses | *Not applicable* |
| Results | | |  |
| Participants | 13* | (a) Report numbers of individuals at each stage of study—eg numbers potentially eligible, examined for eligibility, confirmed eligible, included in the study, completing follow-up, and analysed | *Response rates are reported* |
|  |  | (b) Give reasons for non-participation at each stage | *This is reported:*  *One agency, from Slovenia, responded that they have no resources to provide the data asked in the survey.* |
|  |  | (c) Consider use of a flow diagram |  |
| Descriptive data | 14* | (a) Give characteristics of study participants (eg demographic, clinical, social) and information on exposures and potential confounders | *Not applicable – these data were not collected* |
|  |  | (b) Indicate number of participants with missing data for each variable of interest | *This is reported in detail in the manuscript and in supplementary files with raw data* |
| Outcome data | 15* | Report numbers of outcome events or summary measures | *Outcome data are reported* |
| Main results | 16 | (*a*) Give unadjusted estimates and, if applicable, confounder-adjusted estimates and their precision (eg, 95% confidence interval). Make clear which confounders were adjusted for and why they were included | *Not applicable* |
|  |  | (*b*) Report category boundaries when continuous variables were categorized | *Not applicable* |
|  |  | (*c*) If relevant, consider translating estimates of relative risk into absolute risk for a meaningful time period | *Not applicable* |
| Other analyses | 17 | Report other analyses done—eg analyses of subgroups and interactions, and sensitivity analyses | *Not applicable* |
| Discussion | | |  |
| Key results | 18 | Summarise key results with reference to study objectives | *Key results are reported* |
| Limitations | 19 | Discuss limitations of the study, taking into account sources of potential bias or imprecision. Discuss both direction and magnitude of any potential bias | *Limitations are reported* |
| Interpretation | 20 | Give a cautious overall interpretation of results considering objectives, limitations, multiplicity of analyses, results from similar studies, and other relevant evidence | *Interpretation is reported* |
| Generalisability | 21 | Discuss the generalisability (external validity) of the study results | *Generalisability is addressed in the Limitations.* |
| Other information | | |  |
| Funding | 22 | Give the source of funding and the role of the funders for the present study and, if applicable, for the original study on which the present article is based | *This is reported:*  *“No extramural funding.”* |

*Give information separately for exposed and unexposed groups.

**Note:** An Explanation and Elaboration article discusses each checklist item and gives methodological background and published examples of transparent reporting. The STROBE checklist is best used in conjunction with this article (freely available on the Web sites of PLoS Medicine at http://www.plosmedicine.org/, Annals of Internal Medicine at http://www.annals.org/, and Epidemiology at http://www.epidem.com/). Information on the STROBE Initiative is available at www.strobe-statement.org.
