# Supplementary material for: Workload and procedures used by European data protection authorities related to personal data protection: a cross-sectional study
Source: BMC Res Notes. 2023 Mar 27;16:41. doi: 10.1186/s13104-023-06308-z (PMC10045515; doi:10.1186/s13104-023-06308-z)
Supplement: Supplementary file 3 — Supplementary Material 3 [file 13104_2023_6308_MOESM3_ESM.docx]

**Supplementary file 3. Raw data collected within the study**

# Contents

[Supplementary table 1. Question 1: What is the procedure of responding to opinion/guidance requests in your authority regarding compliance with GDPR and data protection legal framework, and in which deadline? 2](#_Toc97551506)

[Supplementary table 2. Question 2: What is the procedure of handling the **complaints** of the citizens, and in which deadline? 7](#_Toc97551507)

[Supplementary table 3. Question 3: Are there multiple options of dealing with the **complaints** (amicable resolution, mediation) and is there a prespecified maximum response time? 13](#_Toc97551508)

[Supplementary table 4. Question 4: If it is possible, please provide us the number of **opinion/guidance requests** sent by **data controllers and processors** regarding compliance with the data protection legal framework for years: 16](#_Toc97551509)

[Supplementary table 5. Question 5. If it is possible, please provide us the number of **opinion/guidance requests and complaints** sent by **data subjects** for years: 20](#_Toc97551510)

[Supplementary table 6. Question 6: How many **opinion/guidance requests and complaints** regarding personal data protection related **specifically to scientific research** your authority received in the period from January 1, 2015 to May 1, 2020? 25](#_Toc97551511)

[Supplementary table 7: Question 7. Regarding **opinion/guidance requests** and **complaints** related to scientific research, how many of those reported from January 1, 2015 to May 1, 2020 were related specifically to **non-medical** research? 27](#_Toc97551512)

[Supplementary table 8. Question 8: Could you please provide us information on how many **cases/complaints reported by the citizens related to violation of their right to personal data protection** went to the court (official misdemeanor proceedings) in the period from January 1, 2015 to May 1, 2020? 29](#_Toc97551513)

[Supplementary table 9. Question 9: Related to the previous question, regarding **complaints** related to **scientific research**, how many of those reported from January 1, 2015, to May 1, 2020, went to the court (official misdemeanor proceedings)? 32](#_Toc97551514)

[Supplementary table 10. Question 10: Does your authority organize GDPR training sessions/education? 34](#_Toc97551515)

[Supplementary table 11. Question 11: If yes, who is the target audience of such GDPR training sessions /education? 37](#_Toc97551516)

[Supplementary table 12. Question 12: Does your authority organize GDPR training sessions /education for the scientific research community? 39](#_Toc97551517)

[Supplementary table 13. Question 13: If your authority does organize GDPR training/education for the scientific research community, how often do you provide such training/education and how many individuals usually attend such training/education? 40](#_Toc97551518)

# Supplementary table 1. Question 1: What is the procedure of responding to opinion/guidance requests in your authority regarding compliance with GDPR and data protection legal framework, and in which deadline?

| **Data protection agency** | **Response** |
| --- | --- |
| Austria | In general, the Austrian Data Protection Authority (Austrian DPA) does not provide informal guidance as legal opinions can only be provided in a formal complaint procedure pursuant to Article 77 GDPR or a formal data protection investigation pursuant to Article 58(2)(b) GDPR.  However, the Austrian DPA is represented in all expert subgroups of the European Data Protection Board (EDPB) pursuant to Article 68 GDPR and contributes to the drafting of opinions and guidelines of the EDPB. |
| Bulgaria | The Bulgarian Commission for Personal Data Protection in generally, responds within one month upon the opinion/guidance requests receipt. The deadline can be extended depending on the matter, considering that the opinions/guidelines are adopted after decision taken by the Chairman and the member at plenary meeting. The plenary meetings usually take place once a week. |
| Croatia | According to the Act on Implementation of the GDPR, at a written request for opinion of a natural or legal person, the Agency shall provide an expert opinion, no later than within 30 days from the day of submission of the request, depending on the complexity of the request.  If, for the provision of expert opinion, it is necessary to involve other bodies in the country or abroad for the purpose of obtaining data or information essential for providing the expert opinion, the deadline for providing the may be extended for another 30 days. |
| Estonia | The requests are handled according to the Estonian Response to Memoranda and Requests for Explanations and Submission of Collective Proposals Act with the deadline of 30 days (can be extended to 60 days). The request do not have to be (digitally) signed. |
| Finland | The Administrative Procedure Act (*434/2003)* lays down provisions on the foundations of good administration and on the procedure to be applied in administrative matters. The Office of the Data Protection Ombudsman of Finland (hereinafter “**the authority**”, “**the office**”) shall, within its competence, provide with advice on dealing with matters and respond to questions and enquiries concerning the compliance with data protection legislation. Advice shall be provided free of charge. Consideration of the matters shall be publicly accessible as set out in the Act on the Openness of Government Activities (*621/1999*).  Simply, there are a few phases in the procedure, including a pendency of the matter, research of the matter and responding to the opinion/guidance requests. An opinion/guidance request can be made by writing a free form letter/mail by the data subjects, controllers, DPOs or processors. Opinion/guidance requests may also be solved orally. The authority gives a telephone service as well. However, the authority provides advice of a general nature meaning that it does not give detailed or binding opinions on individual issues, unless otherwise decided in an individual case. Accordingly, opinions/guidance requests cannot be judicially reviewed by the appealing courts (administrative courts).  In practice, a specialist shall peruse, resolve, and respond to the opinion/guidance requests independently. With the Data Protection Ombudsman and two Deputy Data Protection Ombudsmen the authority has approximately 40 specialists. Significant opinions/guidance requests shall be resolved together with the (deputy) Data Protection Ombudsman, unless otherwise specified in an individual case. Matters shall be considered without undue delay as set out in the Section 23 of the Administrative Procedure Act. |
| Greece | *The Hellenic Data Protection Authority (HDPA), based on art. 57 par. 1 e of the GDPR, has the authority to provide, upon request, information to the data subjects regarding the exercise of their rights, as they derive from the GDPR. Only the data subjects can submit a request for information. The request must relate exclusively to the exercise of their rights.*  *The HDPA is trying to meet its obligations as soon as possible, but due to the burden of the cases it handles, its answers are not always immediate. The GDPR does not specify the time within which the Authority must respond in writing.*  *Moreover, according to the decision of the HDPA 52/2018 (available on its website), the Authority has no longer the obligation to answer the questions and requests of the controllers, the data subjects and third parties, regarding issues of processing personal data that do not fall under the provisions of the GDPR.*  *The Authority also advises, in accordance with the law, the national parliament, the government and other bodies and organizations on legislative and administrative measures related to the protection of the rights and freedoms of individuals against processing. This is done by issuing the relevant opinions and instructions to the controllers*. |
| Italy | The procedure and deadline for responding to requests for opinions/guidelines from our authority regarding compliance with GDPR is indicated in the following table:   \| **Procedure and legislation** \| **Deadline** \| \| --- \| --- \| \| Opinion following prior consultation following a data protection impact assessment (Article 36, paragraph 1, of the GDPR and Sections 2-p and 110, paragraph 1 of Legislative Decree no. 196/2003). \| 8 weeks after receipt of the request, which can be extended by a further 6 weeks. \| \| Opinions on proposals for legislative measures or regulatory measures based on such legislation (Article 36, paragraph 4, of the GDPR and Section 154, paragraph 5, of Legislative Decree no. 196/2003). \| 45 days after receipt of the request, without prejudice to shorter periods provided for by law. \| \| Opinion on medical, biomedical and epidemiological research programmes and projects (Article 36, paragraph 1, of the GDPR and Section 110, paragraph 1 of Legislative Decree no. 196/2003. \| 8 weeks after receipt of the request, which may be extended by a further 6 weeks. \| |
| Latvia | Generally, the procedure of responding to requests is defined in the Administrative Procedure Law. Regarding opinion/guidance requests, Inspectorate bases on Article 98 of the Law, which provides rights to a statement regarding persons rights. This article determines that private person has the right to receive a statement regarding her rights in a specific legal situation. It must also be added, that such statement is not binding to the addressee of this statement.  The deadline of response is defined in the Article 5 of the Law on Submissions, that stipulates, that institution shall provide a reply on the merits within a reasonable time period, taking into account the urgency of addressing the issue referred to in the submission, but not later than within one month after receipt of the submission, unless otherwise provided by the law.  More general requests for opinion/guidance made by for example a association or a group of private persons are taken into account and provided (or included in the work plan of the Data State Inspectorate) if there is enough resources. |
| Lichtenstein | Requests for opinions or guidance reach us by telephone, e-mail or online-form. They are answered by our team as they come in and as quickly as possible, usually within days. There is no precise deadline. |
| Lithuania | Please be informed that procedure of responding to the request for consultation is set in Article 10 of Law of Republic of Lithuanian on public administration stating that decision on request (which includes consultations to be provided) during 20 working days (please find it here (in Lithuanian only): <https://www.e-tar.lt/portal/lt/legalAct/TAR.0BDFFD850A66/asr>). Also, it is set in Description of consultations, approved by order of director of State Data Protection Inspectorate of the Republic of Lithuania (hereinafter – SDPI) (please find it here: <https://www.e-tar.lt/portal/lt/legalAct/1e99041041a311e99a17eaa929142a91>). |
| Norway | Answer:  The NO SA has a telephone based advisory service, where anyone with guidance requests can call us. The phone line is open for two and a half hours Monday–Thursday. We now have nine law and technology students employed, who facilitate the advisory service along with legal advisors and technologists from the different departments of our supervisory authority.  During 2019, we increased the opening hours for the phone line and stopped the advisory service by e-mail that we offered earlier. The reason behind this change was to increase our capacity, to be able to respond to a higher number of guidance requests in a faster manner. The requests are usually answered during the phone call or subsequently the same day/the day after.  In addition, we produce, translate and publish guidance documents on our website about different parts of the legal framework. Anyone can access information there on the specific area their question relates to, and in this way, we can reach and help more people and businesses.  We sometimes also host guidance meetings for controllers/processors that request our guidance, where we prioritize controllers/processors that process large amounts of personal data or special categories of personal data. |
| Romania | The Romanian DPA does not have a specific procedure for responding to opinion/guidance requests. However, please note that the handling of petitions is regulated, at national level, by Ordinance no. 27/2002 on the regulation of the activity of solving petitions. |
| Slovakia | Slovak SA provides answer on every question from either private or public sector. We do not officially call them “opinion” or “guidance,” but the answer provides guidelines for data subject, controller, processor or DPO. We do not have any legal deadlines for providing answer. For example, some emails/letters are responded within 1 month, other with more complex problem are responded within 6 months.  On our website, we publish disclaimer for lawyers (<https://dataprotection.gov.sk/uoou/sk/content/otazky-advokatskych-kancelarii-adresovane-uradu>), which quotes some lines from document COMMUNICATION FROM THE COMMISSION TO THE EUROPEAN PARLIAMENT AND THE COUNCIL Stronger protection, new opportunities - Commission guidance on the direct application of the General Data Protection Regulation as of 25 May 2018  *“The data protection authorities are the natural interlocutors and first point of contact for the general public, businesses and public administrations for questions regarding the Regulation. The data protection authorities' role includes informing controllers and processors of their obligations and raising the general public’s awareness and understanding of the risks, rules, safeguards and rights in relation to data processing. It does not mean, however, that controllers and processors should expect to be provided by the data protection authorities with the kind of tailored, individualised legal advice that only a lawyer or a data protection officer can provide.”*  As regards to national guidelines, we are not obliged to issue guidelines upon requests. If we decide to issue guidelines, we do not have any legal deadline.  Slovak SA provides also face-to-face consultations and telephone consultation (the latter provided until 2018). However, this is not taken into account in the answer to question 5. |

# Supplementary table 2. Question 2: What is the procedure of handling the **complaints** of the citizens, and in which deadline?

| **Data protection agency** | **Response** |
| --- | --- |
| Austria | For complaint procedures, the Austrian DPA is obliged to respect the Austrian AVG in which national procedural rules are stipulated.  The Austrian AVG stipulates that complaints must be handled within a deadline of six months after the complaint was lodged with the Austrian DPA. In general, after investigating the subject matter of a complaint the procedure ends with a formal decision (ruling) of the Austrian DPA.  However, this deadline can be extended in certain cases, for example when the One-Stop-Shop mechanism pursuant to Article 60(1) GDPR applies. |
| Bulgaria | The complaints handling procedure is set in Section II “Handling Complaints under Article 38 of PDPA and Alerts, Including under Cooperation Mechanism with Other Supervisory Authorities” of the Rules of Procedure of CPDP and its administration, available in English on the following link: <https://www.cpdp.bg/en/index.php?p=element&aid=36>  The general requirements for requests (including complaints) handling are set in Art. 28 of the Rules of Procedure, namely, the requests should contain:  particulars of the requesting party:  - names, mailing address and permanent address, contact telephone number, email address (if available);  - the nature of the request;  - date of knowledge of the infringement, if an infringement is alleged;  - identification of a person targeted by the request;  - other information or documents, where this is provided for in a law or in these Rules;  - date and signature.  The deadline can vary depending on the scope and complexity of the complaint/alert submitted to the Commission. In general, the deadline is 3 months. The handling period will be significantly extended if the case has international outreach and should be handled in accordance with Chapter VII of the GDPR, namely “Cooperation and consistency”. |
| Croatia | Anyone who considers that any of his or her rights guaranteed by this Act and the General Data Protection Regulation have been violated, may submit to the Agency a request for determination of a violation of a right. The Agency shall decide on the violation of rights by a ruling. The ruling of the Agency shall be an administrative act. No appeal shall be allowed against the ruling of the Agency, but an administrative dispute may be instituted by lodging a complaint before a competent administrative court.  In cases where an investigation is conducted, the official person shall, at the request of a Party, issue a decision and submit it to the party no later than 60 days from the date of submission of the orderly request. |
| Estonia - **new** | *The complaint has to be (digitally) signed and fulfill certain requirements (we have published model form on our webpage). The deadline of handling complaints is 30 days, which can be extended additionally for 60 days.* |
| Finland | Simply, the procedure of the complaints consists of the following phases: i) a pendency of an administrative matter; ii) examining the matter and hearing the views of parties; iii) deciding the matter; and iv) a service of notice. The decisions given by the authority can be judicially reviewed by the administrative courts.  In practice, a specialist (a responsible referendary) shall examine the complaints independently. A complaint may be made by writing free form or using a complaint form. The complaints shall indicate what the matter is about and contain the name of the sender and the contact details needed for dealing with the matter. Significant matters shall be examined and resolved together with the (deputy) Data Protection Ombudsman, unless otherwise specified in an individual case. All decisions on a complaint shall be given by the (deputy) Data Protection Ombudsman together with the specialist. Decisions on imposing administrative fines shall be given by the Sanctions Board. The Board is formed by the Data Protection Ombudsman and two deputy data protection ombudsmen and chaired by the Data Protection Ombudsman.  Complaints shall be considered without undue delay as set out in the Section 23 of the Administrative Procedure Act. |
| Greece | *According to article 57 par. 1f of GDPR, the ΗDPA investigates, to the extent appropriate, the subject matter of every complaint. Consequently, the extent in which every complaint is examined depends on the DPA’s judgement.*  *More specifically, the HDPA examines the following complaints that fall within its competence:*   1. ***Violation*** *of data subjects’ rights provided in articles 15 to 22 of GDPR under the condition that the data subject* ***has already exercised her/his rights to the controller,*** *where applicable, and either she/he hasn’t received a reply within the time frame provided for in the article 12 par. 4 of GDPR (one month, with a conditional two month extension) or the reply she/he received by the controller is not satisfactory.* 2. ***Unlawful processing*** *of their personal data, which is violating the provisions for the protection of personal data (GDPR, law 3471/2006 for the protection of personal data in e-communications) and provided that they have appealed to the controller and their issue hasn’t been resolved.*   *Also, the subscriber or user affected, regardless if she/he is a natural person, has the right to submit a complaint in these cases:*   1. ***Violation*** *of provisions for conducting phone calls for the* ***promotion of goods or services*** *(art. 11 par. 1 and 2 of law. 3471/2006), and* 2. ***Violations*** *of provisions for* ***sending unsolicited promotional e-mails*** *(art. 11 law 3471/2006), as e-mail and sms.*   *The complainants submit their complaints by filling in a different form for each case. In every form there are mandatory fields depending on the case. If these are not filled, the DPA has the right not to examine the case regardless of the way a form is used and the required information is not provided for.*  *A complaint is submitted: a) by the data subject, or b) by nonprofit bodies or organizations or unions or associations without legal status that have been established and operate lawfully and the protection of rights and freedoms of data subjects, with regard to the protection of personal data, is mentioned in their statutory objectives, following an assignment by the data subject.*  *The data subject must have exercised, to the controller, the rights provided for in articles 15-22 of the GDPR, where applicable, and either she/he hasn’t received a reply from the controller within the time frame/deadline provided for in article 12 par. 4 of the GDPR or her/his reply is not satisfactory. If the aforementioned procedure, is not followed, the Hellenic DPA doesn’t examine the complaint. Complaints that are vague, unsubstantiated, are submitted abusively, especially due to a repetitive pattern, are filed anonymously or do not include the required information may be deemed inadmissible (archived) by the Hellenic DPA.*  *According to article 77 par. 2 of GDPR, the Hellenic DPA informs the complainant on the progress and outcome of the complaint. More specifically, the HDPA has the obligation within three months of the complaint’s submission to inform the complainant on the progress of the filed complaint. This is especially the case when further investigation is needed or coordination with another supervisory authority. In light of these, the above mentioned time period should not be perceived as the time frame within which the case will be resolved.*  *The procedure of handling the complaints is the following: a) the data subject files the complaint b) within three months of the filing of the complaint the HPDA shall send a document to the person against whom the complaint was filed, requesting his views on the complaint, which shall also be communicated to the complainant, c) it then examines the complaint by issuing a relevant decision. For complaints that are unfounded, inadmissible, vague or abusive, an archiving act is issued, which is signed by the President of the Authority and the competent auditor and which is notified to the complainant.* |
| Italy | Section 8 et seq. of the of the Garante’s Regulation 1/2019, provide that the complaint must be accompanied by a description of the circumstances of the case regarding the violation of the relevant legal provisions and is followed as a rule by a preliminary investigation whilst the subsequent formal administrative procedure may lead to the adoption of the measures referred to in Article 58 of the Regulation. An electronic form is provided on the Garante’s website to facilitate lodging of a complaint.  The Garante shall decide the complaint within nine months from the date of submission; in any case, it shall inform the data subject of the progress or the outcome of the proceedings within three months from that date. In the presence of investigational requirements, to be evidenced and communicated to the data subject by the Garante, the complaint may be decided within twelve months. In case of activation of the procedure of cooperation referred to in Article 60 of the Regulation, the running of time is suspended for the duration of the aforementioned procedure. The decision by the Garante on a complaint may be challenged before judicial authorities according to Section 143 and 152 of the data protection Code and article 78 of the Regulation.  The lodging of the complaint is free of charge. |
| Latvia | **Answer:**  Handling complaints of the citizens the Inspectorate applies two procedures.  **1^st^ procedure**  The first procedure is applied in the case with no clear indication that infringement of data subject’s rights of freedoms has been committed and case is initiated to establish that. Such procedure is regulated by Administrative Procedure Law and Law on Submissions.  Law on Submissions stipulates the procedures by which a private person shall submit and an institution what implements state administration tasks shall examine a document, which includes a request, complaint, proposal or enquiry within the competence of the institution, and shall reply, as well as prescribes the procedures by which the institution shall receive visitors.  The submission may be submitted in writing, in electronic form or expressed in oral form. The submission expressed in oral form shall be drawn up in writing, if necessary, in the presence of a private person and its copy shall be issued to the submitter.  The deadline of response, as mentioned in the previous answer, is defined in the Article 5 of the Law on Submissions, that stipulates, that institution shall provide a reply on the merits within a reasonable time period, taking into account the urgency of addressing the issue referred to in the submission, but not later than within one month after receipt of the submission, unless otherwise provided by the law.  The Administrative Procedure Law also regulates this procedure. In Article 64 are defined time periods for issuing of administrative acts. It stipulates, that if an administrative case is initiated on the basis of a submission, an institution shall take a decision regarding the issue of an administrative act or termination of the matter within a month from the day the submission is submitted, provided that a shorter term is not prescribed in a regulatory enactment.  If due to objective reasons it is not possible to comply with the one month time period, the institution may extend it for a period not exceeding four months from the day the submission is submitted, notifying the submitter thereof. If a lengthy determination of facts is necessary, the time period for taking a decision may be extended for up to one year pursuant to a reasoned decision of the State secretary of the ministry or the head of the local government administration, but if the institution is not subordinated to the Cabinet, the head of the institution, notifying the submitter thereof. The decision regarding extension of the time period may be disputed and appealed. The decision of a court may not be appealed.  In urgent cases, the submitter may apply to the institution with a substantiated submission and request that time period for the issue of the administrative act be abbreviated. The institution shall examine such submission without delay and take a decision in writing. In the event of refusal, the decision shall be notified to the submitter without delay. Such decision may be disputed and appealed.  **2^nd^ procedure**  The second procedure is applied in the case with clearly visible indications that infringement of data subject rights has been committed and sanction can be imposed.  This procedure is regulated by the Latvian Administrative Violations Code. This Code determines, which action or inaction shall be acknowledged as an administrative violation, and what administrative sanction, by which institution (official) and in accordance with which procedures may be imposed upon a person who has committed an administrative violation.  Article 270 of this Code defines time periods for the adjudication (dealing with) of administrative violation matters. It stipulates, that matters regarding administrative violations shall be adjudicated within a period of 30 days from the day when the institution, which is competent to adjudicate the matter, has received the report regarding the administrative violation and other materials of the matter. If due to objective reasons it is not possible to observe the time period of 30 days, the time period may be extended, but not for longer than 30 days.  It must be mentioned that handling complaints of the citizens the Inspectorate also considers time periods determined in the Article 78 (2) of GDPR. |
| Lichstenstein | Official complaints by citizens are filed either by letter, e-mail or through an electronic form we provide on our website. We confirm receipt of it and if the complaint is well founded and not dismissed, we start the investigation of the case which ends with a decision. There is no precise deadline for this, as the length of the proceedings depends on the complexity of the case or its investigation. But of course, every complainant has the right to timely and efficient proceedings. |
| Lithuania- **new** | Please be informed that procedure of complaint handling is explained in Articles 17, 23 – 31 of Law of Republic of Lithuania on Legal Protection of Personal Data (unofficial English translation can be found here: <https://vdai.lrv.lt/en/legislation>, full text in Lithuanian: <https://www.e-tar.lt/portal/lt/legalAct/TAR.5368B592234C/asr>) and in the description of the procedure approved by the director of SDPI (this description can be found here: <https://www.e-tar.lt/portal/lt/legalAct/fb1d82207b4f11eb9601893677bfd7d8>).  Article 30(2) of Law of Republic of Lithuania on Legal Protection of Personal Data states that “The complaint or part thereof must be examined and answered to the applicant within 4 months from receipt of the complaint by the supervisory authority, except in cases where it is necessary to extend investigation of the complaint or part thereof due to the complexity of the circumstances indicated in the complaint or in the part thereof, as well as the scope of information, avoidance of compliance with the requirements of the supervisory authority by the person complained against and other legal or natural persons, continuation of the actions complained against or other objective reasons. In these cases, the time limit for consideration of the complaint or part thereof is extended, but not longer than for 2 months. The general time limit for examination of the complaint or part thereof may not be longer than 6 months from the date of receipt of the complaint by the supervisory authority”. |
| Norway | The NO SA handles all complaints we receive that fall with the scope of Article 77 GDPR (cf. also Article 80). We do not have a maximum procedural deadline for the handling the complaint in national administrative law, but the law states that the case must be handled and decided without undue delay. In cases that will involve a decision decisive for rights and duties for specific physical or legal persons, we are obliged to provide a temporary answer within one month. As of today, our average time for processing a case is approximately six months. For cross-border cases, we calculate a longer processing time. |
| Romania | By Decision no. 133/2018, published in the Official Journal of Romania no. 600 of the 13^th^ of July 2018, the Procedure for handling complaints was adopted. The English version of the Procedure is available on the website of the Romanian DPA at the following link <https://www.dataprotection.ro/index.jsp?page=procedura_plangerilor>. |
| Slovakia | Our national Personal Data Protection Act is available in English language https://dataprotection.gov.sk/uoou/sites/default/files/2019_10_03_act_18_2018_on_personal_data_protection_and_amending_and_supplementing_certain_acts.pdf#overlay-context=sk/content/182018#overlay-context=sk/content/182018%22  Procedure of handling complaints is regulated in Chapter five The Office -> TITLE IV PERSONAL DATA PROTECTION PROCEEDING (sections 99 to 106) of Personal Data Protection Act  Deadlines are regulated under section 101 of Personal Data Protection Act  Section 101  Periods  (1) The Office shall decide in the proceeding within 90 days from the day proceeding is initiated. In the reasonable cases this period is extended by Office, to a maximum of 180 days. The Office notifies the parties of the proceeding about the extension in writing.  (2) If it is necessary to carry out an inspection during the proceeding, the period for issuing a decision pursuant to paragraph 1 does not run, from the day of the inspection is initiated until the inspection is finalised.  (3) If the Office knows that the conditions were met for suspension of the proceedings pursuant to a special regulation,48) the Office shall suspend the proceedings and inform the parties thereof. |

# Supplementary table 3. Question 3: Are there multiple options of dealing with the **complaints** (amicable resolution, mediation) and is there a prespecified maximum response time?

| **Data protection agency** | **Response** |
| --- | --- |
| Austria | There are cases when no formal decision (see question 2) is required, for example when a controller (the opponent in a procedure) fulfills the data subject’s request pursuant to Article 12(3) GDPR before the end of the complaint procedure and the data subject does not object or when the data subject withdraws the complaint. |
| Bulgaria | The Bulgarian Data Protection Authority has supervision competences under the GDPR and the Personal Data Protection Act and other specific national laws. When deciding on the measures that need to be taken after establishing violation of data protection rights are taken into account the Administrative Procedure Code (for complaints) and the Administrative Infringements and Sanctions Act (for alerts). Under Art. 20 of the APC there is a possibility for settlement between the parties of the complaint (Art. 42 of the Rules of Procedure).  In Section III “Application of Measures under Article 58 (2) of Regulation (EU) 2016/679 and Items 3, 4 and 5 of Article 80 (1) and Chapter Nine of PDPA” of the Rules of Procedure are described the rules for issuing measures on complaints/alerts. |
| Croatia | No, maximum response time prescribed by the law is 60 days. |
| Estonia - **new** | *No, we do not have amicable resolutions in Estonia.* |
| Finland | The parties may reach an amicable settlement if the controller complies with the data subject’s request, and the data subject has no additional claims against the controller. It should be noted that the amicable settlement occurs only between the parties in the case. The supervisory authority shall continue pro-ceedings from its own initiative e.g. if there are other similar complaints, the complaint reveals infringe-ments, or the complaint has an impact on the rights and freedoms of other data subjects.  There is not a prespecified maximum response time. Complaints shall be considered without undue delay as set out in the Section 23 of the Administrative Procedure Act. |
| Greece | *Although there is no formal “amicable settlement” provision in our legislation, in practice we use a similar process, when the infringement is a minor one and the data subject is satisfied, so as to resolve large numbers of complaints.* |
| Italy | Section 10(3) of the Garante’s Regulation 1/2019 provides that once the complaint has been received, the Garante verifies whether it is substantiated as for the alleged violations and the measures requested by the applicant. To this end, the Garante examines the documentation received and can acquire clarifications and information regarding the facts and circumstances to which the complaint refers, also by hearing, personally or through an attorney, the data controller or data processor as well as by issuing requests for information or the provision of documents. In this context, the Garante may invite the controller or processor to voluntarily comply with the requests made by the complainant and to communicate this to the Garante within the deadline set by the latter. |
| Latvia | Dealing with complaints, the Inspectorate evaluates the impact of infringement and, in case of low risks to data subject’s rights and freedoms, initially invites the data controller to take all steps to adjust their politics and data processing to the GDPR and national regulation. Just in case, if complainant and data controller doesn’t find satisfactory reconciliation, the Inspectorate proceeds with imposing corrective measures.  At the same time, it must be mentioned that in 2014 Mediation Law in Latvia was adopted, thus creating a legal framework for one of the alternative dispute resolution methods - mediation.  Mediation Law defines mediation as structured co-operation process on voluntary basis whereby the parties attempt to reach a mutually acceptable agreement on the settlement of their dispute with the assistance of a mediator.  Mediation may be used for the settlement of disputes in pretrial proceedings, as well as in judicial proceedings, if it is not stipulated otherwise in the special legal norms.  The parties have a right to decide freely on their participation in mediation, commencement of mediation, selection of a mediator, the course of mediation within limits determined by the mediator, discontinuation and termination of mediation with or without entering into an agreement. |
| Lichstenstein | Whenever possible and feasible, we try to solve cases by amicable resolution or mediation between the parties involved. If successful, the complainant is encouraged to withdraw his complaint so we do not have to decide the case formally anymore. |
| Lithuania- **new** | Currently amicable resolution, mediation is possible to use according to the Article 32-33 of Description of the procedure for complaint handling mentioned above. Even though there are multiple options for resolving complaints, but if complaint is received, SDPI must follow the requirements of national legislation. SPDI has a possibility to go through mediation but if the data subject rejects such option, SPDI must continue the complaint handling procedure leaving the mediation procedure behind.  It is worth to mention, that there was a case in Lithuania, when SDPI used Article 57(4) of GDPR and refused to start the complaints handling procedure because of a huge number of complaints on the same matter but decided to start investigation against the same data controller on its initiative. |
| Norway | Norwegian law does not have the concept of amicable resolution or mediation for dealing with complaints in the field of data protection. |
| Romania | The tasks of the Romanian DPA are regulated by Article 57 of the Regulation (EU) 2016/679, where Article 1 letter f) provides for the task to handle complaints lodged by a data subject, or by a body, organisation or association in accordance with Article 80, and investigate, to the extent appropriate, the subject matter of the complaint and inform the complainant of the progress and the outcome of the investigation within a reasonable period, in particular if further investigation or coordination with another supervisory authority is necessary. The Regulation does not refer to amicable resolution, mediation and, therefore, the Romanian DPA does not have such a task. With reference to the time-limits for handling the complaints, Article 21 paragraph (2) of Law no. 102/2005 on the set up, organisation and functioning of the National Supervisory Authority for Personal Data Processing, republished, provides that “*The national supervisory authority shall inform the data subject about the admissibility of the complaint, within 45 days from the registration. If it is found that the information in the complaint or the documents transmitted are incomplete or insufficient, the National Supervisory Authority requests the data subject to complete the complaint in order to be considered admissible for the purpose of carrying out an investigation. A new deadline of no more than 45 days starts from the date of filing the complaint*.”  Furthermore Article 21 paragraph (3) of the same law states that “*The National Supervisory Authority shall inform the data subject about the progress or outcome of the investigation, within three months from the date on which it was notified that the complaint is admissible in accordance with paragraph (2). The information will also include the remedy against the National Supervisory Authority.*”  At the same time, please note that Chapter III of the Procedure for handling complaints regulates the conditions for analysing and solving complaints. |
| Slovakia | Decisions are regulated under sections 102 and 103 of Personal Data Protection Act.  Maximum response time is regulated under section 101 of Personal Data Protection Act. (see answer to question 2) |

# Supplementary table 4. Question 4: If it is possible, please provide us the number of **opinion/guidance requests** sent by **data controllers and processors** regarding compliance with the data protection legal framework for years:

2015

2016

2017

2018 (in the period from January 1, 2015, to May 24, 2018, pre-GDPR period)

2018 (in the period May 25, 2108, to December 31, 2018, post-GDPR period)

2019

2020 (until May 1, 2020?)

| **Data protection agency** | **Response** |
| --- | --- |
| Austria | There is no such statistic.  Occasionally, the Austrian DPA receives requests to provide a legal opinion on specific cases, but such an opinion cannot be given (see question 1). |
| Bulgaria | 2015- in general 92 opinions  2016- in general 125 opinions  2017- in general 88 opinions  2018 (in the period from January 1, 2015, to May 24, 2018, pre-GDPR period)- 3 opinions on health/ medical data processing, in general 36 opinions  2019- in general 67 opinions  2020- in general 56 opinions |
| Croatia | This are numbers for opinion/guidance request both from data controller/processors and citizens, we don’t have statistical data specially for controllers/processors and specially for citizens.  2015 613  2016 604  2017 850  2018 (in the period from January 1, 2015, to May 24, 2018, pre-GDPR period) 859  2018 (in the period May 25, 2108, to December 31, 2018, post-GDPR period) 2605  2019 1406  2020 (until May 1, 2020?) 432 |
| Estonia - **new** | 2015 *1369*  2016 *1417*  2017 *1520*  2018 (in the period from January 1, 2015, to May 24, 2018, pre-GDPR period) -  2018 (in the period May 25, 2108, to December 31, 2018, post-GDPR period) -  2018 (*full year, most extensive period for requests were a few month before 25 May 2018, but we did not separate the statistics) 2384*  2019 *2343*  2020 (full year) *1759*  *Please note that Estonian Data Protection Inspectorate is supervisory authority in data protection, freedom of information and spam matters. Statistics includes all these areas. We also have helpline to answer simpler requests. The statistics about the number of calls are under the title of “Kõned valveametnikule” in our statistics overview.*  *Our full statistics is available at here:* [*https://www.aki.ee/et/teavitus-uudised/statistika*](https://www.aki.ee/et/teavitus-uudised/statistika) *(year 2020 will be added in short future)* |
| Finland | Between 2015 and May 2018 (pre GDPR) it is not possible to provide exact numbers of opinion/guid-ance requests sent both by **data controllers and processors**. However, the approximate numbers of opinion/guidance requests concerning all sectors sent by **data controllers** are as follows:   - • 2015: circa (hereinafter “c.”) 650 - • 2016: c. 650 - • 2017: c. 700 - • 2018 (*in the period from January 1,* ***2018****, to May 24, 2018*): c. 450   Between 2018 and May 2020 (post GDPR) the approximate numbers of opinion/guidance requests con-cerning all sectors sent both **by data controllers and processors** are as follows:   - • 2018 (*in the period May 25, 2018, to December 31, 2018, post-GDPR period*): c. 600 - • 2019: c. 600 - • 2020 (*until May 1, 2020*) c. 200   *Please notice that the recording practice of the matters has varied, therefore the numbers of the matters are only indicative and not exact.* |
| Greece | *The* ***total number of queries that were submitted to the Hellenic DPA by data controllers/processors AND data subjects*** *regarding the lawfulness of a particular processing or how to implement the relevant legislation in 2015 was 1299. Similarly, in 2016 it was 1465 and in 2017 it was 1751.*  *Τhe* ***total number of queries submitted to the Hellenic DPA by data controllers/processors AND data subjects in 2018 was 1111****.*  *In 2019 826 queries were submitted and 248 in 2020 (until May 1).*  ***Please note that due to the limitations of the current information system, the queries that were submitted by data controllers/processors can’t be separated from the ones submitted by data subjects.*** |
| Italy | **The replies to questions 4, 5, 6 and 7 of the questionnaire have been grouped in a single table. (located below this one)** |
| Latvia | Unfortunately, we can’t provide such information, as the Inspectorate doesn’t collect its statistical data in such distinction. |
| Lichstenstein | 2015 – 476  2016 – 464  2017 – 434  2018 (in the period from January 1, ~~2015~~ 2018, to May 24, 2018, pre-GDPR period) – 405  2018 (in the period May 25, 2018, to December 31, 2018, post-GDPR period) – 1363  2019 – 1647  2020 (until May 1, 2020?) – 332 |
| Lithuania- **old data copied** | In 2015 we received 4.037 requests for consultations by data controllers and processors;  In 2016 we received 4.368 requests;  In 2017 we received 4.459 requests;  In 2018 (pre-GDPR period) we received 3.292 requests;  In 2018 (post-GDPR period) we received 1.942 request;  In 2019 we received 2.896 requests;  In 2020 (until May 1, 2020?) we received 1.151 requests. |
| Norway | Answer:  **2015:**  Total –  Controllers and processors – N/A  **2016:**  Total –  Controllers and processors – 4 684  **2017:**  Total –  Controllers and processors – 5 525  **2018:**  Total – 11 971 (Approximately 70 % of these we received in the period pre-GDPR, and approximately 30 % in the period post-GDPR)  Controllers and processors – 7 183  **2019:**  Total – 7 186  Controllers and processors – 3 737  **2020 (until May 1, 2020):**  Total – Approximately 1786  Controllers and processors – N/A |
| Romania | 2015 – 137  2016 – 409  2017 – 380  2018 (in the period from January 1, 2018, to May 24, 2018, pre-GDPR period) – 128  2018 (in the period May 25, 2108, to December 31, 2018, post-GDPR period) – 820  2019 – 506  2020 (until May 1, 2020?) – 123 |
| Slovakia | See Q5 |

Table with data from Italy for questions 4, 5, 6, and 7

| **File type** | **Grand total of files**  **1 Jan 2015 - 1 May 2020** | **Research files** | **Of which:** | | |
| --- | --- | --- | --- | --- | --- |
|  |  |  | **Medical research** | **Scientific research** | **Research in general** |
| Opinion | 245 | 1 |  |  | 1 |
| Requests for guidance | 1.759 | 15 | 4 | 2 | 9 |
| Complaint (post-GDPR) | 4.084 | 0 |  |  |  |
| Complaint (cases before the entry into force of the GDPR) | 1.013 | 1 | 1 |  |  |
| **Totali** | **7.101** | **17** | **5** | **2** | **10** |

# Supplementary table 5. Question 5. If it is possible, please provide us the number of **opinion/guidance requests and complaints** sent by **data subjects** for years:

2015

2016

2018 (in the period from January 1, 2015, to May 24, 2018)

2018 (in the period May 25, 2108, to December 31, 2018)

2019

2020 until May 1, 2020?

| **Data protection agency** | **Response** |
| --- | --- |
| Austria | Complaints by data subjects on a national-only level (not taking into account data protection investigations initiated by the Austrian DPA and international complaints subject to the One-Stop-Mechanism):  2015: 479 (147 formal complaints and 332 so called “ombudsman procedures”)  2016: 520 (180 formal complaints and 340 so called “ombudsman procedures”)  2017: 489 (156 formal complaints and 333 so called “ombudsman procedures”)  2018: 1159 (1036 formal complaints and 123 so called “ombudsman procedures”; please note such “ombudsman procedures” do not exist anymore since the GDPR)  2019: 2102  2020: no statistic yet  For more detailed statistics see the “Datenschutzbericht” (annual reports), available at:  <https://www.dsb.gv.at/download-links/dokumente.html> |
| Bulgaria | 2015- in general 92 opinions, 817 complaints  2016- in general 125 opinions, 670 complaints  2017- in general 88 opinions, 485 complaints  2018 (in the period from January 1, 2015, to May 24, 2018)- 3 opinions on health/ medical data processing, in general 36 opinions, 780 complaints  2019- in general 67 opinions and 1600 complaints  2020- in general 56 opinions, 680 complaints |
| Croatia | 2015 537  2016 417  2017 524  2018 (in the period from January 1, 2015, to May 24, 2018) 319  2018 (in the period May 25, 2108, to December 31, 2018) 907  2019 1312  2020 until May 1, 2020? 384 |
| Estonia - **new** | 2015 *446*  2016 *390*  2018 (in the period from January 1, 2015, to May 24, 2018) -  2018 (in the period May 25, 2108, to December 31, 2018) -  2018 (full year) *462*  2019 *462*  2020 until May 1, 2020?  2020 (full year) *609*  *Please see explanations from the previous answer* |
| Finland | The approximate numbers of opinion/guidance requests and complaints concerning all sectors sent by data subjects are as follows:   - • 2015: c. 1500 - • 2016: c. 1650 - • 2017: c. 1550 - • 2018 (*in the period from January 1,* ***2018****, to May 24, 2018, pre GDPR*): c. 700 - • 2018 (*in the period May 25, 2018, to December 31, 2018, post GDPR period*): c. 1900 - • 2019: c. 2200 - • 2020: (*until May 1, 2020*): c. 650   *Please notice that the recording practice of the matters has varied, therefore the numbers of the matters are only indicative and not exact.* |
| Greece | ***For the number of queries that were submitted to the Hellenic DPA by data subjects AND data controllers/processors in the aforementioned years please see our answer to question 4.***  ***The number of complaints sent by data subjects*** *in 2015 was 506. Similarly, in 2016 it was 715, in 2017 it was 921, in 2018 841 and 990 in 2019.*  *339 complaints were submitted in 2020 (until May 1).* |
| Italy | See table in the Q4 |
| Latvia | Unfortunately, we can’t provide such information, as the Inspectorate doesn’t collect its statistical data in such distinction. |
| Lichstenstein | 2015 – 116  2016 – 82  2017 – 70  2018 (in the period from January 1, ~~2015~~ 2018, to May 24, 2018) – 44  2018 (in the period May 25, 2018, to December 31, 2018) – 70  2019 – 119  2020 until May 1, 2020? – 59 |
| Lithuania- **new** | 2016 – 5.369 (4.368 of them were requested by data controllers/data processors).  2017 – 5.696 (4.459 of them were requested by data controllers/data processors).  2018 (in the period from January 1, 2015, to May 24, 2018, pre-GDPR period) – 3003 (2.624 of them were requested by data controllers/data processors).  2018 (in the period May 25, 2108, to December 31, 2018, post-GDPR period) – 3.295 (2.610 of them were requested by data controllers/data processors).  2019 – 4.568 (2.835 of them were requested by data controllers/data processors, 61 – by data protection officers)  2020 (until May 1, 2020?) – 2.202 (1.372 of them were requested by data controllers/data processors, 61 – by data protection officers).  Please be informed that numbers above include written answers and phone consultations. |
| Norway | Answer:  **2015:**  Total –  Data subjects – N/A  **2016:**  Total –  Data subjects – 4 851  **2017:**  Total –  Data subjects – 4 899  **2018:**  Total – 11 971 (Approximately 70 % of these we received in the period pre-GDPR, and approximately 30 % in the period post-GDPR)  Data subjects – 4 788  **2019:**  Total – 7 186  Data subjects – 3 449  **2020 (until May 1, 2020):**  Total – Approximately 1786  Data subjects – N/A |
| Romania | 2015 – 66 (requests); 1074 (complaints)  2016 – 122 (requests); 2014 (complaints);  2017 – 144 (requests); 3543 (complaints)  2018 (in the period from January 1, 2018, to May 24, 2018) – 17 (requests); 1900 (complaints)  2018 (in the period May 25, 2108, to December 31, 2018) – 460 (requests); 2922 (complaints)  2019 – 254 (requests); 5808 (complaints)  2020 until May 1, 2020? – 167 (requests); 1791 (complaints) |
| Slovakia | We do not keep records/statistic for controller, processor, data subject, DPO separately for each category. Therefore, numbers below inform about any **opinion/guidance** we provide notwithstanding of the subject/body by email/letter as explained in answer to question 1.  2015 **450**  2016 **520**  2017 **788**  2018 (in the period from January 1, 2018, to May 24, 2018) **348**  from May 25, 2018 to December 31, 2018 **1481**  2019 **1327**  2020 until May 1, 2020? **335**  **Proceedings based on complaints lodged by data subject**:  2015 **136**  2016 **89**  2017 **59**  2018 (in the period from January 1, 2018, to May 24, 2018) **37**  from May 25, 2018 to December 31, 2018 **45**  2019 **68**  2020 until May 1, 2020? **29** |

# Supplementary table 6. Question 6: How many **opinion/guidance requests and complaints** regarding personal data protection related **specifically to scientific research** your authority received in the period from January 1, 2015 to May 1, 2020?

| **Data protection agency** | **Response** |
| --- | --- |
| Austria | There is no such statistic. |
| Bulgaria | **General remark 1:**  Personal data processing in health sphere is regulated by various national legal acts, the main of which is the Health Act, where are included also the medical and non-medical researches.  **General remark 2:**  No specification of the received opinion/request and complaints were made so the provided number are general and no divided by scientific/non-medical research indicator.  For 2015- 1 opinion, generally where submitted 817 complaints on which were performed 687 inspections (prior, on-going and post) of which 276 concerned the healthcare sector and 2 concerning scientific-research activity.  For 2016- generally where submitted 670 complaints, 712 inspections were performed including on cases in 2015 (prior, on-going and post) of which 266 concerned the healthcare sector.  For 2017- 1 opinion (genome research) and 2 in healthcare sphere, generally where submitted 485 complaints, 938 inspections were performed including on cases in 2016 (prior, on-going and post) of which 460 concerned the healthcare sector.  For 2018- 3 opinions on health/ medical data processing, generally where submitted 784 complaints, 303 inspections were performed including on cases in 2017 (prior, on-going and post) of which 115 concerned the healthcare sector.  For 2019- 1 opinion on data controller-processor capacity in medical, clinical researches- 1600 complaints were submitted in general, 955 inspections were performed, including on cases in 2018.  For 2020- in general 680 complaints, 144 inspections were performed. |
| Croatia | 42 |
| Estonia - **new** | *We do not held specific statistics about it, but the probably the number of complaints is very minimal (almost none). The practice about requests is much higher, because the Inspectorate have an obligation to give permission for scientific researches in certain areas (pre-GDPR period: for all scientific researches which fall under the national Personal Data Protection Act, after-GDPR period: only scientific researches conducted for political developments).* |
| Finland | Between 2015 and May 2018 it is not possible to provide numbers of opinion/guidance requests and complaints regarding merely **scientific research**. However, after May 2018 (post GDPR) matters re-garding scientific research are as follows:   - • 2018: 49 - • 2019: 67 - • 2020-May 2020: 16   (together 132 matters)  *Please notice that the recording practice of the matters has varied, therefore the numbers of the matters are only indicative and not exact.* |
| Greece | *39 such queries were submitted and no complaint.* |
| Italy | See table in the Q4 |
| Latvia | According to our data, in this period we have **not received** such requests or complaints. |
| Lichstenstein | Genealogy (ancestry research): < 10 |
| Lithuania- **new** | Regarding **Q. 6–7 & 9**, please be informed that SDPI does not have separate statistic on requests/complaints related to scientific or other research. |
| Norway | Unfortunately, we cannot provide you with a specific answer to this question. Without being able to provide any specific numbers, we know that there have been few complaints with this topic.  In the period between February 1^st^ 2020 – June 19^th^ 2020, we have had 1766 guidance requests in total, where 64 fell within the newly introduced category “research and higher education”. |
| Romania | We do not have such statistics based on this criterion. |
| Slovakia | We do not keep records/statistic about this. |

# Supplementary table 7: Question 7. Regarding **opinion/guidance requests** and **complaints** related to scientific research, how many of those reported from January 1, 2015 to May 1, 2020 were related specifically to **non-medical** research?

| **Data protection agency** | **Response** |
| --- | --- |
| Austria | There is no such statistic. |
| Bulgaria | For 2015- generally where submitted 817 complaints on which were performed 687 inspections (prior, on-going and post). 276 inspection concerned the healthcare sector and 2 handled scientific-research activity.  For 2016- generally where submitted 670 complaints, 712 inspections were performed including on cases in 2015 (prior, on-going and post) of which 266 concerned the healthcare sector.  For 2017- generally where submitted 485 complaints, 938 inspections were performed including on cases in 2016 (prior, on-going and post) of which 460 concerned the healthcare sector.  For 2018- 3 opinions on health/ medical data processing, generally where submitted 784 complaints, 303 inspections were performed including on cases in 2016 (prior, on-going and post) of which 115 concerned the healthcare sector.  For 2019- in general- 1600 complaints were submitted in general, 955 inspections were performed, including on cases in 2018.  For 2020- in general- 680 complaints- 144 inspections |
| Croatia | 34 |
| Estonia - **new** | *We do not collect specific statistics about it. From our previous answer you can see the change of some conditions – at the moment we give permissions only for the policy studies (and these are non-medical). Of course it doesn’t mean that data subject is not allowed to ask or complain about other types of studies.* |
| Finland | It is not possible to provide numbers of opinion/guidance requests and complaints regarding only non-medical research. |
| Greece | *30 such queries were submitted.* |
| Italy | See table in the Q4 |
| Latvia | According to our data, in this period we have **not received** such requests or complaints. |
| Lichstenstein | Genealogy (ancestry research): < 10 |
| Lithuania- **new** | Regarding **Q. 6–7 & 9**, please be informed that SDPI does not have separate statistic on requests/complaints related to scientific or other research. |
| Norway | Unfortunately, we cannot provide you with a specific answer to this question. Without being able to provide any specific numbers, we know that there have been few complaints with this topic.  In the period between February 1^st^ 2020 – June 19^th^ 2020, we have had 1766 guidance requests in total, where 64 fell within the newly introduced category “research and higher education”. |
| Romania | We do not have such statistics based on this criterion. |
| Slovakia | We do not keep records/statistic about this. |

# Supplementary table 8. Question 8: Could you please provide us information on how many **cases/complaints reported by the citizens related to violation of their right to personal data protection** went to the court (official misdemeanor proceedings) in the period from January 1, 2015 to May 1, 2020?

| **Data protection agency** | **Response** |
| --- | --- |
| Austria | If this question refers to court complaints against formal decisions of the Austrian DPA, then from 2015 to 2019 the number is 312.  At the moment of answering this questionnaire, the annual report for 2020 is in process so there is no statistic yet.  If this question refers to other court cases not associated with the Austrian DPA, we have no such statistic. |
| Bulgaria | For 2015- 63 cases went to court- first instance- Sofia district Administrative Court and 37 went to the second instance court- Supreme Administrative Court.  For 2016- 59 cases went to court- first instance- Sofia district Administrative Court and 57 went to the second instance court- Supreme Administrative Court.  For 2017- 44 cases went to court- first instance- Sofia district Administrative Court and 46 (from previous years as well) went to the second instance court- Supreme Administrative Court.  For 2018- 44 cases went to court- first instance- Sofia district Administrative Court and 42 (from previous years as well) went to the second instance court- Supreme Administrative Court.  For 2019- 90 cases went to court- first instance and 32 went to second instance- Supreme Administrative Court.  For 2020- 64 cases went to first instance court and 52 to second instance, including cases from 2019. |
| Croatia | No such data |
| Estonia - **new** | *We do not held that kind of information. Please note that data subject has a right to directly turn to court, without previous contacts with data protection authority.* |
| Finland | Information on how many cases/complaints, reported by the citizens related to violation of their right to personal data protection went **to the court** (official misdemeanor proceedings) in the period from January 1, 2015 to May 24, 2018 is not available. However, the authority is aware of the following matters filed to **the administrative courts** before May 25, 2018 (pre GDPR):   - • 2015: 18 - • 2016: 14 - • 2017: 15 - • 2018/May: 4   **(together 51)**  The authority is aware of the following matters filed to **the courts** after May 25, 2018 (post GDPR):   - • 2018: 18 - • 2019: 14 - • 2020: 7   (together 39)  *Please notice that the recording practice of the matters has varied, therefore the numbers of the matters are only indicative and not exact.* |
| Greece | *From January 1, 2015 to May 1, 2020, 23 decisions were subject to appeal before the Council of State (Supreme Administrative Court of Greece).* |
| Italy | 550 proceeding relating to infringement of personal data legislation, of which 411 appeals against Garante’s decisions. |
| Latvia | During this period 124 cases were sent to the court. |
| Lichstenstein | None |
| Lithuania- **new** | Please be informed that SDPI decision were challenged to courts 75 times in 2015 – 2020 (till April 30). |
| Norway | In addition to proceedings before national courts, in Norway, parties to a case can appeal to the Privacy Appeals Board, which is an independent administrative court-like body that has the competence to review decisions made by the Norwegian SA (although not in cross-border cases).  In total  In the period from January 1, 2015 to May 1, 2020, only one decision made by the Norwegian SA/the Privacy Appeals Board was brought before national courts. |
| Romania | We do not have such statistics based on this criterion. |
| Slovakia | We do not keep records/statistic for controller, processor, data subject, DPO separately for each category. Therefore, numbers below inform about any **opinion/guidance** we provide notwithstanding who brought it to the court.  2015 **2**  2016 **4**  2017 **6**  2018 **6**  2019 **3**  2020 until May 1, 2020? **2** |

# Supplementary table 9. Question 9: Related to the previous question, regarding **complaints** related to **scientific research**, how many of those reported from January 1, 2015, to May 1, 2020, went to the court (official misdemeanor proceedings)?

| **Data protection agency** | **Response** |
| --- | --- |
| Austria | Not applicable |
| Bulgaria | **General remark:** No specification of the sent cases/complaints was made so the provided number are general and no divided on scientific/non-medical research indicator.  For 2015- in general 63 cases went to court- first instance- Sofia district Administrative Court and 37 went to the second instance court- Supreme Administrative Court.  For 2016- in general 59 cases went to court- first instance- Sofia district Administrative Court and 57 went to the second instance court- Supreme Administrative Court.  For 2017- 44 cases went to court- first instance- Sofia district Administrative Court and 46 (from previous years as well) went to the second instance court- Supreme Administrative Court.  For 2018- 44 cases went to court- first instance- Sofia district Administrative Court and 42 (from previous years as well) went to the second instance court- Supreme Administrative Court.  For 2019- 90 case went to court- first instance and 32 went to second instance- Supreme Administrative Court.  For 2020- 64 cases went to first instance court and 52 to second instance, including cases from 2019. |
| Croatia | Not applicable |
| Estonia - **new** | *We do not held that kind of information, but as far as we know there were not any that kind of cases.* |
| Finland | Information on how many complaints related to scientific research went to the court (official misde-meanor proceedings) is not available. However, the authority is not aware of any such complaints. |
| Greece | Not applicable |
| Italy | 20 cases were related to scientific research. |
| Latvia | None of them were related to scientific research. |
| Lichstenstein | None |
| Lithuania- **new** | Regarding **Q. 6–7 & 9**, please be informed that SDPI does not have separate statistic on requests/complaints related to scientific or other research. |
| Norway | None.  In the period January 1, 2015, to May 1, 2020, one decision based on a complaint related to scientific research was appealed before and decided by the Privacy Appeals Board. |
| Romania | We do not have such statistics based on this criterion. |
| Slovakia | We do not keep records/statistic about this. |

# Supplementary table 10. Question 10: Does your authority organize GDPR training sessions/education? Yes/No

**Question 10: Table with categorization of responses for questions 10 and 11**

| **Data protection agency** | **Q10 Response** | **Q11. Response** |
| --- | --- | --- |
| Austria | No | Not applicable |
| Bulgaria | Yes | DPOs |
| Croatia | Yes | -SMEs  -DPOs from all sectors  -Children |
| Estonia - **new** | Yes | -Data subjects  -Data controllers  -Data processors  -Media |
| Finland | Yes | -General public  -Various authorities  -Companies  -DPOs |
| Greece | Yes | -DPOs  -Civil servants  -Lawyers  -General public  -Researchers  -Children |
| Italy | Yes | -DPOs  -SMEs  -Legal professions |
| Latvia | Yes | -Public sector  -Private sector  -Children  -SMEs |
| Lichstenstein | Yes | -Communal authorities  -DPOs  -Associations  -General public  -Students and their parents  -Certain groups of professionals |
| Lithuania- **new** | Yes | -DPOs  -Journalists  -Start-ups  -Representatives of healthcare services  -SMEs  -Vulnerable society groups  -Youth  -Seniors |
| Norway | No | Not applicable |
| Romania | No | Not applicable |
| Slovakia | No | Not applicable |

Acronyms: DPO=data protection officer, SME=small and medium enterprises

**Question 10: All responses, as received**

| **Data protection agency** |  |
| --- | --- |
| Austria | No, but at the moment we take part in the EU project “privacy4kids”.  This is an EU-funded project where we work on providing children-friendly information concerning data protection and privacy together with the University of Vienna. |
| Bulgaria | Yes |
| Croatia | YES |
| Estonia - **new** | *Yes, in some extent* |
| Finland | Yes |
| Greece | Yes  *The Hellenic DPA does organize or coorganize GDPR training sessions/education.*  *More particularly, the HDPA in cooperation with the National School of Public Administration and the Greek Bar Associations have organized several such seminars. Also,*  *the Hellenic DPA, organized training seminars (1-2 March and 29-30 March 2019) for Data Protection Officers of the health sector in the context of the “Problem-based training on the data protection reform package in GR and CY — TRAIN-GR-CY” project, carried out in partnership with the Centre of European Constitutional Law — Institute of Themistocles and Dimitris Tsatsos (coordinator), the Laboratory of Law and Informatics of the National and Kapodistrian University of Athens in Greece and the Office of the Commissioner for Personal Data Protection in Cyprus and the University of Cyprus.*  *It also organizes seminars in collaboration with the Hellenic Scientific Council for the Information Society.* |
| Italy | Yes, although not on a regular basis, but so far mainly as part of EU-funded projects |
| Latvia | **Answer:** Yes  The Inspectorate on a regular basis organizes seminars (available also as a paid service) about GDPR issues.  The seminars are organized on such topics - personal data protection, registration of personal data processing, personal data protection audit, video surveillance, implementation of GDPR and other personal data protection issues. |
| Lichstenstein | Yes |
| Lithuania- **new** | Yes |
| Norway | We do not organize GDPR training sessions/education ourselves, but we do participate with training sessions/education in events organized by others, e.g. businesses or organizations. |
| Romania | Due to the limited resources the Romanian DPA did not organized training sessions.  However, please note that, within the period 2015-2020, the Romanian DPA has organised different conference, e.g. conferences dedicated to the European Data Protection Day celebrated on the 28^th^ of January, organised each year, series of events dedicated to the celebration of one year since the application of Regulation (EU) 2016/679.  At the same time, the representatives of the Romanian DPA has actively participated in the most important events in the field of data protection, organised by various public institutions or private entities, including non-governmental organizations and also participated in a series of radio and TV interviews. |
| Slovakia | Every year, Slovak SA organises event on Data Protection Day. Otherwise, our Slovak SA does not organize GDPR training, but our employees are often host speakers on different GDPR workshop/training etc. organized by different subjects or bodies.  Data Protection Day 2020  We are organising an interactive workshop (open for public), during which we will be presenting various data protection topics. After presentations, there will be possibility to ask questions and discuss with employees of our Office.  Programme:   1. Opening Speech of the President of SK DPA 2. Monitoring via Video Surveillance Systems Based on the Latest Findings of the European Data Protection Board 3. Security and Data Breach Notifications 4. Data Protection Proceedings with International Aspect 5. Frequently Asked Questions about various Data Protection Issues 6. Discussion |

# Supplementary table 11. Question 11: If yes, who is the target audience of such GDPR training sessions /education?

| **Data protection agency** | **Response** |
| --- | --- |
| Austria | Not applicable |
| Bulgaria | In general, the target audience varies depending on the CPDP’s practice and received requests for training.  There can be specific trainings encompassing the new GDPR provisions- for example- DPOs training. |
| Croatia | Small and medium enterprises, DPOs from public sector and private sector including education, health sector and DPOs from all sectors, children. |
| Estonia - **new** | *Data subjects, data controllers, data processors, media etc.* |
| Finland | It may vary. The target audience of such GDPR training sessions may be e.g. the public, different com-munities/unions/associations, other authorities, companies, and data protection officers. |
| Greece | *So far the target audience of these GDPR sessions/education has been data protection officers, civil servants (students of National School of Public Administration), lawyers, the general public, researchers and children* |
| Italy | Within the T4Data project, co-financed by the European Commission, in 2018 and 2019, local training seminars were organised in four different Italian regions with a special focus on DPOs in the public sector thanks also to the collaboration of local authorities; each seminar dealt with a distinct area, offering the opportunity for in-depth analysis and direct comparison through questions and answers from users: Ancona (The processing of personal data for health care and research purposes); Catanzaro (The protection of personal data and transparency of the Public Administration after Regulation (EU) 2016/679); Turin (Risk management and security of processing); Rome (Liability for processing and fines).  In addition, webinars for public sector DPOs have been provided, through a dedicated web-based platform, in collaboration with officials and managers from the Garante, divided into the following four modules for a total of more than 40 hours of lessons: 1) The fundamentals of data protection; 2) Role and skills of the DPO in the public sector; 3) The DPO toolkit; 4) Sectoral insights (health care, scientific research, schooling, public administration). Also available to users are the slides used for each webinar.  Within the SMEDATA project, co-financed by the European Commission, which aims to ensure the effective application of the General Regulation on the Protection of Personal Data through awareness raising, multiplication of training and sustainable capacity development for SMEs and legal professions, in 2019, in collaboration with the Roma Tre University, 12 regional training events were organized in 6 Italian cities; senior and junior staff from the Garante contributed as speakers along with university professors.  In order to develop a proposal of criteria for the construction of a self-assessment tool that aims to support SMEs of different sizes and belonging to different economic sectors in assessing their level of compliance with the GDPR, two workshops were organized in October, where the document containing the draft criteria for the construction of the self-assessment tool was presented. The workshops were attended by managers and officials of the most important Italian SME trade associations. |
| Latvia | There are representatives from the public and private sector, pupils and high-school students in audience.  It must be mentioned, that the Inspectorate from December of 2018 implements a project "General Data Protection Regulation – Opportunities ad Responsibility for Small and Medium-sized Enterprises (SMEs); Rights and Risks for Minors" (DPSME) within the framework of the Rights, Equality and Citizenship Financial Programme for 2014–2020. During the implementation of the project Inspectorate organizes seminars for small and medium-sized entrepreneurs and also for minors aged 13-17. |
| Lichstenstein | We have made GDPR training sessions / education for communal authorities, for data protection officers, for associations, for the general public, for students and their parents and for certain groups of professionals. |
| Lithuania- **new** | The target audience depends on concrete training. For example, currently SDPI it is planning to have trainings for data protection officers on issues that they meet in their work.  SDPI has also carried out the project SolPriPa (Solving privacy paradox) during which numerous training were provided for different target audiences: journalist, start-ups, representatives of health care services, small and medium enterprises, vulnerable society groups, youth, seniors. More about the project (in Lithuanian): <https://vdai.lrv.lt/lt/naudinga-informacija/solpripa-projektas>, some information can also be found here: <https://vdai.lrv.lt/en/news/solpripa-project-successfully-implemented>  This year SDPI will also carry out the project SolPriPa WORK which aim is provide training to employers and employees on work related data protection issues. |
| Norway | Depending on the type of session/education, the audience can be controllers or processors in both private and public sector, data protection officers and lawyers/legal advisors. |
| Romania | If we are talking about the conferences organised by the Romanian DPA, the target audience is the public and private sector, general public. |
| Slovakia | Data Protection Day event is open for public- everybody is welcome, no matter what sector (public/ private) |

# Supplementary table 12. Question 12: Does your authority organize GDPR training sessions /education for the scientific research community?

| **Data protection agency** | **Response** |
| --- | --- |
| Austria | No |
| Bulgaria | 2018- CPDP has carry out training on the GPDR application with 19 participants (16 of which were from various universities).  The training topics were:  - general data protection requirements in the GDPR;  - practical steps that need to be taken by the data controllers in order to implement the new data protection standards;  - the risks, rules, measures and rights of individuals subject to personal data processing  - personal data processing and standards in the scientific field. |
| Croatia | No |
| Estonia - **new** | *It is possible and it depends on their request for the training.* |
| Finland | It may organize. Recently, the authority of Finland has not organized GDPR training sessions /education for the scientific research community. |
| Greece | *So far the HDPA hasn’t organized GDPR training sessions* ***especially for the scientific research community****. Yet, it is noted that a number of researchers does attend the seminars the HDPA is organizing in collaboration with the Hellenic Scientific Council for the Information Society.* |
| Italy | Not yet, but senior and junior staff from the Garante participate regularly, as speakers, in various conferences concerning the protection of personal data in the field of scientific research. |
| Latvia | Until now the Inspectorate has not organized GDPR training sessions for the scientific research community. |
| Lichstenstein | Some of the above mentioned sessions were held at universities. Even if they were not specifically targeted at scientific researchers, they were open for them to attend as well. |
| Lithuania- **new** | SDPI has not organized trainings for the scientific research community specifically. |
| Norway | See answer to question number 10. Our legal advisors and technologists do participate in training sessions/education by holding workshops/presentations for the scientific research community. The frequency depends on the interest/demand in the community. |
| Romania | No |
| Slovakia | No, so far we have not organised such event. |

# Supplementary table 13. Question 13: If your authority does organize GDPR training/education for the scientific research community, how often do you provide such training/education and how many individuals usually attend such training/education?

| **Data protection agency** | **Responses** |
| --- | --- |
| Austria | Not applicable |
| Bulgaria | About 1 per year with about 20-50 participants. |
| Croatia | We organize education for DPOs from all sectors, and also DPOs and controllers from scientific research community can participate. In 2021 we started with counducting trainings for DPOs every month, for approximately 600 DPOs. |
| Estonia - **new** | *Most of our trainings are initiated by the audience, we haven’t conducted any trainings for researchers during last couple of years.* |
| Finland | Not applicable |
| Greece | *The frequency and the number of individuals that attend these sessions vary (the range for the latter is from 20-25 to 170).* |
| Italy | *No response* |
| Latvia | Not applicable |
| Lichstenstein | We have not organized GDPR training / education specifically for the scientific research community (see answers above). Therefore, we do not know how many researchers have attended our other trainings / education. |
| Lithuania- **new** | Not applicable |
| Norway | Without the ability to provide any official numbers, the number of sessions/workshops/presentations since 2015 seem to be allocated like this:  **2020: 0**  **2019: 5**  **2018: 0**  **2017: 7**  **2016: 4**  **2015: 2**  Usually participants range from 20 to 50, but this can vary.  Keep in mind, these are sessions/education organized by others where we have participated. |
| Romania | Not applicable |
| Slovakia | Not applicable |
